# Alternative reproductive strategies and the maintenance of female color polymorphism in damselflies

**DOI:** 10.1002/ece3.3083

**Published:** 2017-06-15

**Authors:** Rosa A. Sánchez‐Guillén, Maren Wellenreuther, Jesús R. Chávez‐Ríos, Christopher D. Beatty, Anais Rivas‐Torres, María Velasquez‐Velez, Adolfo Cordero‐Rivera

**Affiliations:** ^1^ Instituto de Ecología AC (INECOL) Red de Biología Evolutiva Xalapa, Veracruz Mexico; ^2^ Department of Biology Lund University Lund Sweden; ^3^ Institute for Plant and Food Research Limited Nelson New Zealand; ^4^ Centro Tlaxcala de Biología de la Conducta Universidad Autónoma de Tlaxcala Tlaxcala Mexico; ^5^ Departamento de Biología Celular y Fisiología Instituto de investigaciones biomédicas Universidad Nacional Autónoma de México Tlaxcala Mexico; ^6^ Department of Ecology & Evolutionary Biology Cornell University Ithaca NY USA; ^7^ ECOEVO Lab Departamento de Ecoloxía e Bioloxía animal Universidade de Vigo Vigo Spain; ^8^ Laboratorio de Zoología y Ecología Acuática (LAZOEA) Universidad de los Andes Bogotá Colombia

**Keywords:** behavior, fecundity, female‐limited color polymorphism, learned‐mate preferences, sexual conflict

## Abstract

Genetic polymorphisms are powerful model systems to study the maintenance of diversity in nature. In some systems, polymorphisms are limited to female coloration; these are thought to have arisen as a consequence of reducing male mating harassment, commonly resulting in negative frequency‐dependent selection on female color morphs. One example is the damselfly *Ischnura elegans*, which shows three female color morphs and strong sexual conflict over mating rates. Here, we present research integrating male tactics, and female evolutionary strategies (female mating behavior and morph‐specific female fecundity) in populations with different morph‐specific mating frequencies, to obtain an understanding of mating rates in nature that goes beyond the mere measure of color frequencies. We found that female morph behavior differed significantly among but not within morphs (i.e., female morph behavior was fixed). In contrast, male tactics were strongly affected by the female morph frequency in the population. Laboratory work comparing morph‐specific female fecundity revealed that androchrome females have lower fecundity than both of the gynochrome female morphs in the short term (3‐days), but over a 10‐day period one of the gynochrome female morphs became more fecund than either of the other morphs. In summary, our study found sex‐specific dynamics in response to different morph frequencies and also highlights the importance of studying morph‐specific fecundities across different time frames to gain a better understanding of the role of alternative reproductive strategies in the maintenance of female‐limited color polymorphism.

## 1 INTRODUCTION

In the last 20 years, an increasing number of studies have investigated the role of sexual conflict and its relevance in the evolution of mating interactions (Arnqvist & Rowe, 2005; Clutton‐Brock & Parker, 1995). Sexual conflict arises when the two sexes have different optimal fitness strategies. This directly affects the mode and frequency of mating, with males typically attempting to mate as often as possible, while females—for whom individual reproductive events are usually more costly—try to minimize the number of matings (Arnqvist & Nilsson, 2000). Sexual conflict over optimal mating strategies can thus select for females to evolve traits that reduce male harassment (Arnqvist & Rowe, 2005). Among these evolved traits are female‐limited polymorphisms (Schluter, 2001), which make it harder for males to form a female search image and thus dilute the overall level of harassment to females (Fincke, 2004).

Damselflies (Odonata: Zygoptera) have been used as a model system in pioneering research on polymorphism because they constitute an extremely rich group of species with a genetic female‐limited color polymorphism (at least 100 species), which is in several cases likely phylogenetically conserved (Fincke, Jödicke, Paulson, & Schultz, 2005) and related to the degree of polygamy (Robinson & Allgeyer, 1996). In particular, the damselfly genus *Ischnura* mostly harbors polymorphic species and has thus become an evolutionary and ecological model system to study the forces maintaining this color diversity (see Wellenreuther, Svensson, & Hansson, 2014). Sexual conflict has been used to explain the maintenance of color polymorphism in damselflies (Sánchez‐Guillén, Hansson, Wellenreuther, Svensson, & Cordero‐Rivera, 2011; Svensson, Abbott, Gosden, & Coreau, 2009). Ischnuran females likely suffer fitness costs from excessive male mating harassment, which reduces foraging time and increases the risk of injuries and predation (Gosden & Svensson, 2007; Takahashi & Watanabe, 2010a). In *Ischnura*, color morphs normally include one morph with a malelike coloration (the “androchrome”) and one or more morphs with colors different from the conspecific male (“gynochromes”). This color polymorphism is controlled by one locus with three alleles with complete and hierarchical dominance (*androchrome *> *infuscans *> *infuscans‐obsoleta*) (Sánchez‐Guillén, Van Gossum, & Cordero‐Rivera, 2005) and governed (in nature) by the combined action of stochastic and selective forces (Andrés, Sánchez‐Guillén, & Cordero Rivera, 2000; Sánchez‐Guillén et al., 2011; Takahashi, Kagawa, Svensson, & Kawata, 2014).

Several hypotheses have been proposed to explain the maintenance of the female‐limited color polymorphism in damselflies. One explanation that has been put forward based on mathematical (Sherratt, 2001) and verbal models (Cordero, 1992; Hinnekint, 1987; Johnson, 1975; Robertson, 1985; Sirot & Brockmann, 2001; Utzeri, 1988; Van Gossum, Stoks, & De Bruyn, 2001b) is that female morphs use color in avoiding male harassment. In line with this is that female color morphs show different mating frequencies (Cordero‐Rivera & Sánchez‐Guillén, 2007; Hammers, Sánchez‐Guillén, & Van Gossum, 2009; Hammers & Van Gossum, 2008), but also different fecundity (Banham, 1990; Iserbyt, Bots, Van Gossum, & Sherratt, 2013; Takahashi & Watanabe, 2010b), aggressive behavior (Sirot & Brockmann, 2001), and parasite prevalence (Sánchez‐Guillén, Martínez‐Zamilpa, Jiménez‐Cortés, Forbes, & Córdoba‐Aguilar, 2013; Willink & Svensson, 2017). A key aspect in understanding the maintenance of the female‐limited color polymorphism in ischnuran damselflies that has thus far, however, been neglected is the possibility that color morphs could be signaling alternative reproductive strategies (see Roulin, 2004; Roulin & Bize, 2006 and references therein). This idea has recently received considerable support, with increasing evidence suggesting that the pigments necessary to produce alternative colors may have pleiotropic effects on physiological attributes (Armbruster, 2002; Eliason, Shawkey, & Clarke, 2016; Forsman, Ringblom, Civantos, & Ahnesjö, 2002; Merrill, Van Schooten, Scott, & Jiggins, 2011; Roulin, Almasi, Meichtry‐Stier, & Jenni, 2011). Such alternative strategies have only been studied in a handful of male‐polymorphic organisms (Ahnesjo & Forsman, 2003; Hutchings & Myers, 1994; Lank, Smith, Hanotte, Burke, & Cooke, 1995; Tuttle, 2003) and in three female‐polymorphic organisms: butterflies (Ellers & Boggs, 2002), fishes (Craig & Foote, 2001), and recently, reptiles (Galeotti et al., 2013). In ischnuran damselflies, evidences for the link between color and behavior come from two studies with *Ischnura ramburii* (Sirot, Brockmann, Marnis, & Muschett, 2003) and *I. elegans* (Van Gossum, Stoks, & De Bruyn, 2001a). Moreover, the link between fecundity and color has been detected in *Ischnura elegans* (Banham, 1990) and *Ischnura senegalensis* (Takahashi & Watanabe, 2010b). However, the lack of studies including both fecundity and behavior in androchrome and gynochrome females from populations with highly differentiated female morph frequencies has prevented an estimation of the role of female morphs in the maintenance of the color polymorphism.

Here, we characterized the role of the alternative reproductive strategies of *I. elegans* females in the maintenance of this female‐limited color polymorphism, due to the fact that this species (1) has a well‐studied female‐limited genetic color polymorphism; (2) possesses color and physiological traits—such as behavior (Van Gossum et al., 2001a) and fecundity—that are correlated (Banham, 1990); and (3) has been shown in our long‐term study populations in Spain to have contrasting equilibrium morph frequencies and variable densities (Cordero‐Rivera & Sánchez‐Guillén, 2007; Sánchez‐Guillén et al., 2005, 2011), allowing us to disentangle social environmental effects (frequency and density). Our prediction is that female behavior and intrinsic fecundity will be linked to each color morph and fixed, that is, will not change between populations, while male behavior, in terms of male preference for the different female morphs, should be plastic, that is, will be molded by the female morph frequency in the population, to avoid losing mating opportunities (Sánchez‐Guillén, Hammers, et al., 2013; Van Gossum, Bruyn, & Stoks, 2005; Van Gossum et al., 2001a). To test this hypothesis empirically, we combine field data and laboratory tests to explore the role of morph‐specific behavior and morph‐specific fecundity, in addition to female color, in modulating female morph mating frequencies in nature for *I. elegans*. Our field data include behavioral population data from 3 years, including the number of single female morphs, the number of female morphs in copula, male preferences for both female morphs (androchrome and gynochrome), and female morph sexual and nonsexual responses to male harassment.

## 2 METHODS

### 2.1 Study species and reproductive behavior

**Figure 1:**
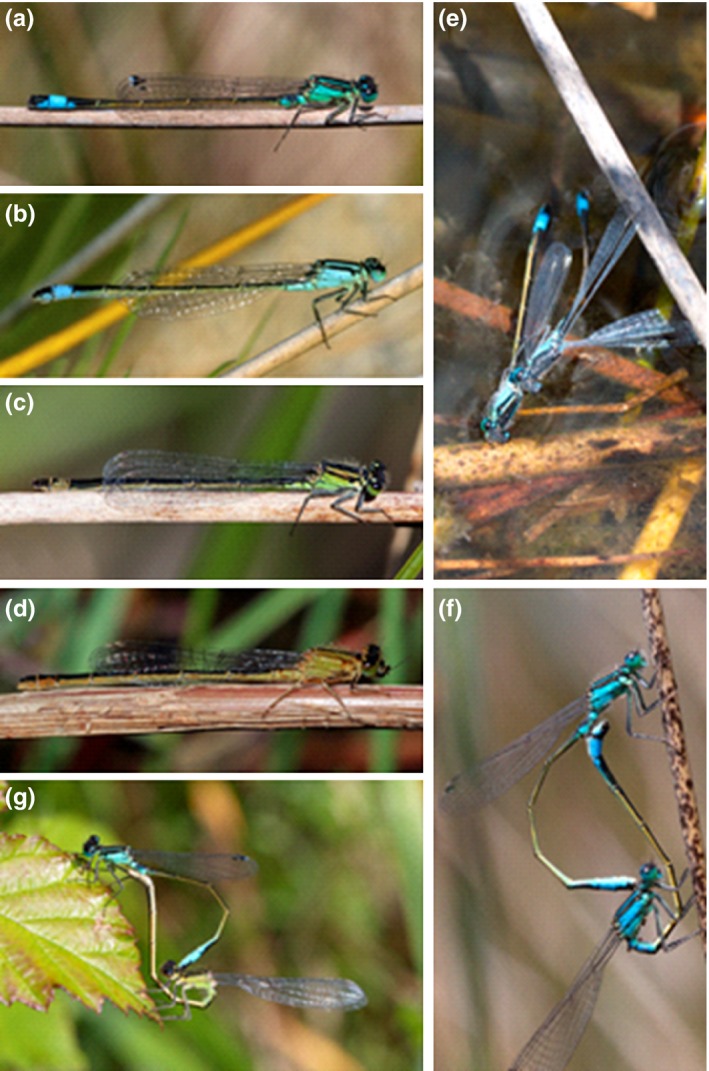
Female color polymorphism in *Ischnura elegans* and basic steps of reproductive behavior. (a) Blue male, (b) blue androchrome female, (c) gynochrome *infuscans* female at green color phase, and (d) gynochrome *infuscans‐obsoleta* female at brown color phase. (e) One androchrome female immobilized by a male, which bites her wings before grasping her in tandem. Both have fallen on the water. (f) Copulation involving one androchrome and (g) one *infuscans* female. Pictures from Laxe population (a, e, f), Louro (b), Xuño (d), and Doniños (g), all in NW Spain, taken by ACR

*Ischnura elegans* is a common damselfly in Europe, with one blue male form (Figure 1a), while mature females occur as one of three discrete morphs: one blue and malelike androchrome morph (Figure 1b) and two green–brown gynochrome morphs, which are called *infuscans* (Figure 1c) and *infuscans‐obsoleta*, respectively (Figure 1d). Although the coloration of the androchrome females and the conspecific males is virtually identical, they can be distinguished by visual examination of external genitalia and the abdomen width (e.g., females have a wider abdomen). Reproductive behavior in this species can be characterized as scramble competition. A male searches for a female, and upon detection, he grasps her (*attempt to tandem*; Figure 1e) by her prothorax using his anal appendages (*tandem*). If the female is receptive, she bends her abdomen upward, and they will form the wheel position and copulate for up to 7 hr (*copula*; Figure 1f,g) (Miller, 1987).

### 2.2 Field morph and mating frequencies of study populations

The experiments were conducted on three of our long‐term study populations (Louro, Laxe, and Doniños) situated in northwestern Spain (see Cordero‐Rivera & Sánchez‐Guillén, 2007) between 2004 and 2015. Androchrome frequencies ranged from (mean over years) 6.5% in Doniños, 47% in Laxe, to 87.5% in Louro. These populations were chosen because they appear to be at equilibrium; that is, female morph frequencies remained similar and stable over multiple years (14 years; two generations per year) (Cordero‐Rivera & Sánchez‐Guillén, 2007; Sánchez‐Guillén et al., 2005; Sánchez‐Guillén, Hammers, et al., 2013).

The frequency of each female morph, the operational sex ratio (only mature adults), and the ratio of males/androchrome females were estimated in all populations using intensive sweep netting over the study area before the period of reproductive activity (09:00–11:00 hr). After this time period (i.e., with increasing ambient temperature), males start to search for suitable females for copulation. Mating frequencies of female morphs were estimated by recording all mating pairs observed in transects performed during the period of reproductive activity (11:00–15:00 hr). Operational sex ratio was estimated as the number of single mature males divided by the number of single mature females found around the pond just before reproductive activity, which are assumed to be receptive to mating.

### 2.3 Experiment 1: Male preference for female morphs

To evaluate male mating preferences for female morphs based on color and behavior (i.e., allowing for behavioral differences such as morph differences in aggression levels), a live‐model presentation experiment was performed during the period of reproductive activity (10:00–15:00 hr) at Laxe and Doniños on three consecutive days in 2013 and 2014. A similar experiment was carried out in Louro in 2001 (Cordero Rivera & Sánchez‐Guillén, 2007). Live models included blue males, blue androchromes, and olive‐green‐to‐brown *infuscans* gynochromes that were all tethered with a fine thread, to allow the model to fly naturally during the course of the experiment (see Cordero, Santolamazza Carbone, & Utzeri, 1998; Cordero‐Rivera & Sánchez‐Guillén, 2007 for detailed methodologies). Each model type was presented until responses from five focal males were recorded. Focal males were subsequently marked whenever possible to avoid testing them twice. Male focal responses were categorized as nonsexual or sexual. The nonsexual categories included *approach* (the male approaches the model closely (<10 cm), but no physical contact is made) and *contact* (the male approaches the model and achieves contact), and the sexual categories included *attempt to tandem* (the male approaches and perches on the model, curving the abdomen in an attempt to achieve tandem) and *tandem* (the male grasps the model with his anal appendages).

### 2.4 Experiment 2: Female morph behavior in response to male harassment

Observations of female morph responses to male mating attempts were recorded during the period of reproductive activity (10:00–15:00 hr) at Louro (2004), Laxe (2007), and Doniños (2013). For each observation, a focal female was chosen at random, and then followed for a period of 13–15 min. During that time, we recorded the maximum and minimum height, estimated by eye, at which the female perched in the vegetation and the percentage of the observational period that the female was “hidden” (perched within dense vegetation where plant stems were at least partially covering the female, so that an approaching male would not see the female silhouette complete). If a male approached, we recorded if the female showed no‐response, or a nonsexual response: *refusal display* (spreading of the wings and curling of the abdomen), *moves around the perch* (to avoid contact with the male), *fly‐away, face‐off* (confronting the approaching male), and *charge* (attacking the approaching male). All interactions between females and males were scored. We also quantified male sexual behavior (*attempt to tandem* and *tandem*) and female sexual behavior (*accepting the copula*). To circumvent potential observer bias, we alternated between androchrome and gynochrome females between each successive observational period. Females were collected after focal observation and marked so that they would not be studied again.

### 2.5 Experiment 3: Intrinsic female morph fecundity

To examine any possibility of an age‐related and/or a morph‐related effect on female fecundity, 40 adult females obtained from rearing in the laboratory (at fifth–sixth day following emergence) were mated, and the day after mating, they were allowed to oviposit. Oviposition containers were made of plastic boxes that were covered with humid filter paper (see Sánchez‐Guillén, Wellenreuther, & Cordero‐Rivera, 2012 for details).

Because the frequency of the *infuscans‐obsoleta* females in our studied populations (Louro, Laxe, and Doniños) was very low (1%–2%), the 40 adult females obtained from rearing eggs in the laboratory come from females captured in five natural populations from Belgium, France, Spain, and Sweden, with high frequencies for the three female morphs. Adult mature females (*N* = 40; 15 androchrome, 20 *infuscans*, and 5 *infuscans‐obsoleta* females) were mated with one male from their own population (they did not suffer male harassment), following established protocols (van Gossum, Sánchez‐Guillén, & Cordero‐Rivera, 2003; Sánchez‐Guillén et al., 2005) and controlling for age (as stated previously, all females were 5–6 days old when mated) and color morph. We estimated morph‐specific fecundity by allowing them to oviposit every day until the third day (*N* = 40; 15 androchrome, 20 *infuscans*, and 5 *infuscans‐obsoleta* females) and until the ninth day (*N* = 24; 9 androchrome, 10 *infuscans*, and 5 *infuscans‐obsoleta*). Fecundity was estimated by counting total eggs i) in the first three clutches and ii) in the first nine clutches.

### 2.6 Statistical analyses

Observed and expected mating frequencies were compared with a χ^2^ test. Data from the three experiments were analyzed using generalized linear models (GLMs).

First, male preference for female morphs was analyzed with a binomial distribution and a logit link function. The response variable was male behavior (sexual response = 1; nonsexual response = 0). The predictor variables were as follows: color morph (androchrome/gynochrome/male); population (Laxe/Doniños); and year (2013/2014), and all main effects and two‐way interactions between predictor variables.

Second, to test whether morph‐specific female behavior is fixed or covaries with female morph frequencies, we used GLMs with a normal distribution and an identity link function where the response variables were the female behaviors *time hidden*, no‐response, *moves around perch, refusal display, fly‐away, charge, or face‐off*, and GLMs with a normal distribution and an identity link function when the response variables were *attempt to tandem*;* tandem*; or *mating*. For all GLMs, the predictor variables were as follows: female morph (androchrome/gynochrome), population (Louro/Laxe/Doniños), and all two‐way interactions between predictor variables.

The most parsimonious models were selected using Akaike's Information Criterion (AIC). We calculated the likelihood of a focal model using AIC weights. When the AIC weight of the best model was <0.9, we used model averaging (Grueber, Nakagawa, Laws, & Jamieson, 2011). For models that gained similar support (ΔAIC < 2), we selected the model with the fewest number of parameters as the most parsimonious model (Burnham & Anderson, 2002).

## 3 RESULTS

### 3.1 Morph and mating frequencies in the field

Androchrome frequencies were highly variable between populations ranging from 4%–89%, but stable among years within populations, ranging from 4%–9% (mean = 6.5%) in Doniños, 44%–50% (mean = 47%) in Laxe, to 86%–89% in Louro (mean = 87.5%) (Table 1). Although the operational sex ratio was similar among populations and years (1.48–4.73; mean = 2.67), the male/androchrome ratio was very different between populations, ranging from 2.58 in Louro to 118.22 in Doniños (mean = 23.41). In two cases, androchrome females were observed to mate statistically less often than expected by their frequency, and in four cases, there was no difference (Table 1). In the seventh case, the androchrome morph was the one that mated more often than its population frequency (Table 1).

**Table 1:** Morph and mating frequencies in the three studied *Ischnura elegans* populations in Spain. Columns show the number of males and females captured, and the operational sex ratio (OSR). Observed and expected mating frequencies were compared with a chi‐square test. A denotes androchrome and I *infuscans* females

Population	Date	N ♀	N ♂	Male/A	OSR	Alone frequencies		Mating frequencies		Chi‐square test	*p*	Source
						A	I	A	I			
Louro	2001	50	111	2.58	2.22	0.86	0.14	0.75	0.15	**14.96**	**.000**	Cordero‐Rivera and Sánchez‐Guillén (2007)
Louro	2004	120	409	4.75	3.41	0.89	0.11	0.82	0.17	**13.24**	**.001**	Cordero‐Rivera and Sánchez‐Guillén (2007 **)**
Laxe	2007	93	163	3.97	1.48	0.44	0.56	0.47	0.53	0.48	.490	This study
Laxe	2013	42	99	4.71	2.36	0.50	0.50	0.45	0.55	0.59	.442	This study
Laxe	2014	104	227	4.37	2.18	0.50	0.50	0.60	0.40	1.65	.198	This study
Doniños	2013	107	506	118.22	4.73	0.04	0.92	0.15	0.85	**36.99**	**.000**	This study
Doniños	2014	58	132	25.29	2.28	0.09	0.90	0.08	0.92	0.01	.926	This study

Significant values are given in bold.

### 3.2 Experiment 1: Male preferences for female morphs

**Figure 2:**
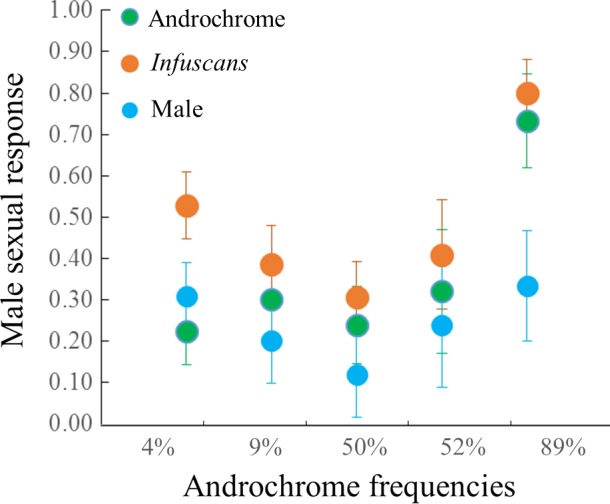
Male mating preferences. Male behavioral responses (mean ± *SE*) to three live models (male, androchrome and gynochrome females) in the three populations ordered by androchrome frequencies: 4% Doniños (*N* = 33); 9% Doniños (*N* = 67); 50% (Laxe 2013; *N* = 61); 52% (Laxe 2014; *N* = 68); and 89% (Louro; *N* = 90) [data from Cordero‐Rivera & Sánchez‐Guillén, 2007]. Males are represented by blue dots, androchrome females by green dots, and *infuscans* females by orange dots

We analyzed male preference of a total of 90 males (data from Cordero‐Rivera & Sánchez‐Guillén, 2007) from Louro (2004), 68 and 61 males from Laxe (2013 and 2014), and 33 and 67 males from Doniños (2013 and 2014) (see Table 2 and Figure 2). Of the seventeen models tested (i.e., from the simplest including one factor, population, model or year, to a model including three factors and all interactions; see models 1–17; Table S1), the model with highest support (Table S1) included two fixed factors: the model presented (androchrome, *infuscans*, or male) and the year (2004, 2013, and 2014). Male preferences were not significantly different toward males or androchrome females (Wald test: χ^2^ = 0.26, *p* = .6093), but were higher toward *infuscans* females (Wald test: χ^2^ = 15.69, *p* < .0001). Male preferences were significantly different between years (Wald test (2014): χ^2^ = 32.18, *p* < .0001; Wald test (2013): χ^2^ = 16.90, *p* < .0001).

**Table 2:** Male preferences for the different female morphs in *Ischnura elegans*. Male focal responses were categorized as nonsexual *approach* (the male approaches the model closely, but no physical contact is made), *contact* (the male approaches the model and achieves contact), and sexual *attempt to tandem* (the male approaches and perches on the model, curving the abdomen in an attempt to achieve tandem) and *tandem* (the male grasps the model with his anal appendages). *N* represents the number of focal males included in each test

Population	*N*	Model	Nonsexual	Sexual	Source
Louro 2004	30	Androchrome	8	22	Cordero‐Rivera and Sánchez‐Guillén (2007)
	31	*Infuscans*	6	25	
	30	Male	20	10	
Laxe 2013	21	Androchrome	16	5	This study
	23	*Infuscans*	16	7	
	17	Male	15	2	
Laxe 2014	25	Androchrome	17	8	This study
	22	*Infuscans*	13	9	
	21	Male	16	5	
Doniños 2013	10	Androchrome	7	3	This study
	13	*Infuscans*	8	5	
	10	Male	8	2	
Doniños 2014	18	Androchrome	14	4	This study
	36	*Infuscans*	17	19	
	13	Male	9	4	

### 3.3 Experiment 2: Female morph behavior to male harassment

A total of 65 androchrome and 69 *infuscans* females were each focally observed for 13–15 min (Table 3). Females that mated with a male during the observation period were removed from the data set (sexual responses) to avoid differences in observation time. The model with highest support (Table S2) explaining the *time hidden* (%) included the population and the morph as fixed factors. *Infuscans* females spend significantly more time (60.42%) hidden than androchrome females (40.24%) (Wald test: χ^2^ = 10.54, *p* = .0012), and additionally, females spend most time hidden in Laxe (Wald test: χ^2^ = 10.93, *p* = .0009). Table S2 shows all model details.

**Table 3:** Female morph behavior in response to male harassment in *Ischnura elegans*. *N* denotes the number of focal females observed for a period of 13–15 min in each population. *Time hidden* shows the percentage of the observed time that the female spent hidden (perched within dense vegetation). If a male approached the female, we recorded the number of nonsexual responses: *refusal display* (spreading of the wings and curling of the abdomen), *moves around the perch* (to avoid contact with the male), *fly‐away*,* face‐off* (confronting the approaching male), and *charge* (attacking the approaching male); and the number of male sexual responses (attempt to tandem and tandem) and female sexual behavior (accepting the mating)

Population	*N*	Female morph	Time hidden (%)	Nonsexual responses					Sexual interactions		
				Spread wings, curl abdomen	Move around perch	Fly‐away	Charge	Face‐off	Attempt to tandem	Tandem	Mating
Louro 2004	7	Androchrome	71.77	4	1	3	3	6	na	na	na
	6	*Infuscans*	90.66	1	2	4	1	2	na	na	na
Laxe 2007	40	Androchrome	29.58	19	2	47	4	42	3	1	1
	39	*Infuscans*	57.95	48	12	50	1	10	3	2	2
Doniños 2013	24	Androchrome	50.52	15	13	20	1	8	11	6	4
	29	*Infuscans*	64.31	24	14	11	0	9	19	14	6

No‐response and two nonsexual responses (*spread* and *face‐off*) were explained by models including only the morph as a fixed factor (Table S2), indicating that these represent fixed differences between morphs. No differences were detected in *no‐response* (Wald test: χ^2^ = 1.74, *p* = .1869) and *spread* between morphs (Wald test: χ^2^ = 3.52, *p* = .0606), while the *face‐off* response was more common in androchrome than *infuscans* females (Wald test: χ^2^ = 5.57, *p* = .0182). The remaining nonsexual behaviors (*move around the perch, fly‐away*, and *charge*) were explained by models including the population as a fixed factor (Table S2). No differences were detected in *fly‐away* (Wald test: χ^2^ = 3.74, *p* = .1537) between populations, while Doniños females responded most frequently with the *moving around the perch* (Wald test: χ^2^ = 4.10, *p* = .0429) and *charging* response (Wald test: χ^2^ = 7.97, *p* = .0186).

Sexual responses by males (*attempt to tandem*,* tandem*, and *mating*) were explained by models including the population as a fixed factor (Table S2). In Doniños, males *attempted to tandem* (Wald test: χ^2^ = 23.35, *p* < .001) and also successfully formed a *tandem* (Wald test: χ^2^ = 3.87, *p* = .0491) more frequently than in Laxe, although females accepted the *copula* in a similar proportion in both populations (Wald test: χ^2^ = 3.48, *p* = .0621).

### 3.4 Experiment 3: Intrinsic components of female morph fecundity

Fifteen androchrome, twenty *infuscans*, and five *infuscans‐obsoleta* females were included in the analysis of the laboratory fecundity data. All females oviposited for at least three consecutive days (*N* = 40), and of these females, 9 androchrome, 10 *infuscans*, and 5 *infuscans‐obsoleta* still managed to oviposit on the ninth day (*N* = 24).

**Figure 3:**
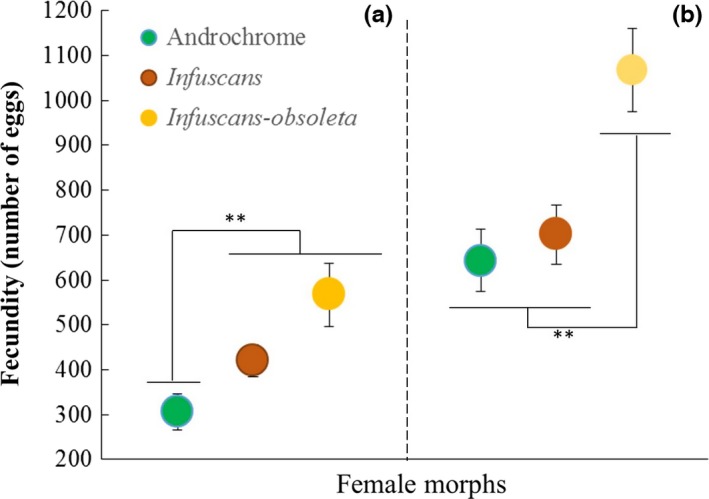
Female fecundity in laboratory. Female fecundity (mean ± *SE*) in the laboratory for (a) the first three clutches and (b) the first nine clutches

When we analyzed the first three clutches, androchrome females laid significantly fewer eggs (306.8 ± 40.14) (Wilcoxon, *Z* = 2.65; *p* = .0232; Figure 3a) than *infuscans* (419.9 ± 34.76), and *infuscans‐obsoleta* females (566.6 ± 69.52; Wilcoxon, *Z* = 2.27; *p* = .0080; Figure 3a). Both gynochrome females (*infuscans* and *infuscans‐obsoleta*) laid a similar number of eggs (Wilcoxon, *Z* = 1.63; *p* = .1029; Figure 3a). However, a different pattern emerged when the first nine clutches were compared: Androchrome females laid significantly fewer eggs (643.3 ± 69.46) (Wilcoxon, *Z* = 2.39; *p* = .0169; Figure 3b) than *infuscans‐obsoleta* females (1067.4 ± 93.19), but a similar (Wilcoxon, *Z* = 1.02; *p* = .3074; Figure 3b) number of eggs to the *infuscans* females (701.10 ± 65.89). Moreover, *infuscans‐obsoleta* females laid a greater number of eggs than the *infuscans* females (Wilcoxon, *Z* = 2.38; *p* = .0239; Figure 3b; Bonferroni‐adjusted *p*‐value (.0253) for multiple comparisons).

## 4 DISCUSSION

Our study presents multiyear field and laboratory data on male and female behavior and female morph‐specific fecundity to obtain an improved understanding of the role of morph‐specific alternative reproductive tactics in the maintenance of a color polymorphism. We found that the most important variable affecting male mating preferences was female morph type: Males showed a clear and consistent preference for gynochrome females, except in cases where androchrome females were the majority morph.

This preference could be explained by different female morph behaviors. For instance, androchromes were more likely to engage in aggressive *face‐offs* than gynochrome females when approached by a male, whereas *infuscans* females were commonly hidden in the vegetation. In fact, previous studies showed that gynochrome females are involved in a higher number of matings than androchrome females (e.g., Sánchez‐Guillén, Hammers, et al., 2013). Given that offspring paternity is almost completely sired by the last male (Cooper, Miller, & Holland, 1996; Sánchez‐Guillén, Córdoba‐Aguilar, & Cordero‐Rivera, 2013), the advantage to males to mate with androchrome females may be to ensure the paternity of a higher number of clutches, although these clutches have a lower number of eggs (in the short term; 3‐day period) than clutches from gynochromes. In contrast to male behavior, females showed a fixed response within but not among morphs, indicating strong sexual differences in the lability of mating behavior. This is consistent with earlier work showing that male morph preferences are developmentally labile and strongly shaped by morph‐specific interactions during ontogeny (Sánchez‐Guillén, Hammers, et al., 2013).

### 4.1 New evidence for the interplay between male mating preferences and female morph frequencies

In a previous study, Sánchez‐Guillén, Hammers, et al. (2013) detected an innate preference in males for androchrome morphs, which is lost after interactions with gynochrome females. Indeed, in natural populations, when androchrome frequency is high, males behaved indiscriminately [Louro (87.5% of androchromes) and Laxe (47% of androchromes)], but preferred the *infuscans* morph when it was the majority morph [Doniños (6.5% of androchromes)] (Cordero‐Rivera & Sánchez‐Guillén, 2007; Sánchez‐Guillén, Hammers, et al., 2013). In our study (with live‐model presentations), we detected that the most important response variable determining male preference is the female morph. Males were more likely to approach the *infuscans* than the androchrome morphs. Additionally, when comparing male preferences for female morphs between populations, males approached *infuscans* more often than androchrome females in all populations, but this preference was only significant in the population (Doniños) where the *infuscans* morph was the majority (91%). In the other two populations, where androchrome females were the majority (84%–89%) or equally frequent (44%–52%), males appeared indiscriminate. It is interesting to note that in only one case, androchrome females mated more frequently than expected by chance (Table 1, Doniños population in 2013); this occurred when the ratio of males/androchromes was over 100 (four to twenty times larger than in the other populations or years), and suggests that when male density is extremely high, even androchromes cannot escape male attention, and consequently, their mating frequency increases. Extreme events like this might have drastic effects on female fitness and fuel stochastic processes in color polymorphism maintenance. Previous work by our group [by genotyping six unlinked microsatellites (Wellenreuther, Sánchez‐Guillén, Cordero‐Rivera, Svensson, & Hansson, 2013)] has shown that the three studied populations are connected by high gene flow (see Wellenreuther, Sánchez‐Guillén, Cordero‐Rivera, Svensson, & Hansson, 2011), and divergent selection and gene flow contribute to the maintenance of the color polymorphism (Sánchez‐Guillén et al., 2011), thus allowing us to discard genetic population differentiation as the underlying cause of morph frequency differences. Thus, selection pressures associated with the interactions of male and female behaviors appear to maintain the long‐term morph frequency differences between these populations.

### 4.2 Interplay between female behavior and mimicry

In recent experiments on mate recognition in the damselfly *Enallagma hageni*, Xu, Cerreta, Schultz, and Fincke (2014) showed that male damselflies implement a set of decision rules based on different morphological cues (namely color and pattern) when selecting potential mates. In this particular species, androchromes are considered to be imperfect mimics of males (they are similar to males in coloration, but resemble gynochromes rather than males in their abdominal patterning). If androchromes indeed minimize male harassment because of their mimetic male resemblance (Cordero, 1992; Fincke, 2004; Hinnekint, 1987; Miller & Fincke, 1999; Robertson, 1985; Sherratt, 2001) then androchromes of *I. elegans* represent a relatively accurate mimic, in that they resemble males in both color and patterning, although androchromes of *I. elegans* still differ in having a wider abdomen than males.

In our study, we used field experiments to explore morph‐specific behaviors as yet another piece in the puzzle to understand the mating dynamics in this species. Previous studies on the damselflies *Nehalennia irene*,* I. elegans*, and *I. ramburii* (Forbes, Schalk, Miller, & Richardson, 1997; Sirot et al., 2003; Van Gossum et al., 2001a) detected that androchrome females tend to be more aggressive compared to gynochrome females, for example, *facing‐off* or *charging* males rather than engaging simply in the refusal display. However, whether morph‐specific behavior is relatively fixed or varies with female morph frequencies was until now unresolved because, in all previous studies, the androchrome females were the minority morph. In our study, we have detected that several behaviors remained relatively unchanged in the three populations (i.e., *time hidden* and *face‐off*), despite the large interpopulation variation in androchrome frequency (Doniños 6.5%, Laxe 47%, and Louro 87.5% of androchrome females). *Infuscans* females spent more *time hidden* than androchrome females, and androchrome females engaged more often than *infuscans* females in *face‐offs* (avoiding matings) when approached by a conspecific male.

### 4.3 Interplay between intrinsic fecundity and mimicry

Studies on birds (e.g., *Strix aluco*; Roulin, Ducret, Ravussin, & Altwegg, 2003), reptiles (e.g., *Lacerta vivipara*; Vercken, Massot, Sinervo, & Clobert, 2007), and insects (e.g., *Ischnura senegalensis*; Takahashi & Watanabe, 2010a, 2010b) have found that reproductive strategies (in terms of number, length or weight of eggs) are correlated with female color polymorphism. Takahashi et al. (2010b) suggested that the androchrome female of the dimorphic *I. senegalensis* is an *r*‐strategist (high fecundity with small eggs), while the gynochrome female morph better represents a *k*‐strategist (low fecundity with large eggs). By contrast, previous studies in *I. elegans* showed that the androchrome females have a lower number of mature eggs in the ovarioles (see Banham, 1990) as well as a lower fecundity (Gosden & Svensson, 2009). However, previous studies failed to detect fecundity differences between the two gynochrome morphs. Our investigations of the intrinsic components of female morph fecundity detected for the first time different strategies not only between androchrome and gynochrome morphs (in the short term), but also between the two nonmalelike gynochrome morphs. *Infuscans‐obsoleta* females were more fecund than either the *infuscans* or androchrome females in the long term, thus revealing different morph‐specific reproductive traits between gynochrome females that contribute to the overall fitness of each female morph.

### 4.4 Male mating preference, female resistance, and reproductive strategies

Our data allowed us to link male‐mating preferences, female morph reproductive strategies, and female morph responses to male harassment to explain a long‐standing question: How is the female color polymorphism maintained in natural populations in terms of male‐mating preferences (i.e., male costs) and different female reproductive strategies (i.e., female costs)? Specifically, our data help to understand why males, even when they have an innate naïve preference for androchrome females, change their preference to gynochrome females following interactions with them, yet behave indiscriminately when androchrome females represent the majority morph. Firstly, gynochrome females are more often *hidden* among vegetation than androchrome females, and this can increase mate‐searching costs for gynochrome females, especially when they are the minority morph. However, when a male detects a female, and this female is an androchrome female, the male is then more likely to encounter an aggressive *face‐off* response. The finding that different female morphs in our study display consistent behaviors across populations with different morph frequencies has interesting implications for understanding the relative mating costs of males. If the behavior of different female morphs is more fixed and thus more canalized, then males in populations with high androchrome frequencies are likely to incur relatively higher mating costs over their lifetime. This is corroborated by our finding (Table 2; Figure 2) that males lose their preference for gynochromes in populations dominated by androchromes and thus that males will approach more androchromes on average. Secondly, androchrome females are less fecund than gynochrome females in the short term (3‐day period); however, this difference is lost between the androchrome and the *infuscans* morph in the long term (9‐day period), although it is maintained by the *infuscans‐obsoleta* females, which always show higher fecundity than the androchrome and the *infuscans* females. Because fecundity is positively correlated with longevity (Leather, 1988), it is important to highlight that no differences in female morph longevities have been detected in *I. elegans* (see Cordero et al., 1998). Thus, males must prefer to mate with *infuscans* and *infuscans‐obsoleta* females even if they are more difficult to find, based on their higher short‐term fecundity. However, if the androchrome female is the majority morph, males must behave indiscriminately, in order to not lose any mating opportunity, even if they are likely to encounter higher female resistance to mating. Based on previous studies on *I. elegans* that have showed that gynochrome females are involved in a higher number of matings than androchrome females (e.g., Sánchez‐Guillén, Hammers, et al., 2013) and that offspring paternity is almost completely sired by the last male (Cooper et al., 1996; Sánchez‐Guillén, Córdoba‐Aguilar, et al., 2013), the advantage to males to mate with androchrome females may be to ensure the paternity of a higher number of clutches (although these clutches have a lower number of eggs than gynochrome clutches).

## 5 CONCLUSION

The dynamic interplay of female mating behavior and morph‐specific fecundity effects may facilitate the occurrence of alternative strategies over time and space, allowing population dynamics to rapidly respond to the prevalent ecological conditions. Males, with their plastic mate preference for female morphs, can rapidly adjust their tactics to the morph frequency in their current population. Females show fixed behaviors associated with their morph, and also predictable differences in morph fecundity. Andromorphs are often the minority morph and tend to have lower short‐term fecundity and also experience lower harassment; gynomorphs tend to have higher fecundity and thus tend to persist as the majority morph in the population. In the specific case of *infuscans‐obsoleta*, this predominantly low‐frequency morph has higher long‐term fecundity, perhaps allowing it to persist in the population. Such dynamics allow different mating scenarios to be adaptive over time. Evidence for a rapid change of male tactics was found when analyzing the androchrome mating rates and male mating preferences across years, both of which varied greatly (see Tables 1 and 2).

Our results therefore show how context‐dependent male behavior, combined with morph‐specific female behavior and fecundity, can combine to maintain long‐term polymorphisms in damselfly populations, even in populations that differ in morph frequencies. These findings have implications for damselflies as well as other taxa. Future studies should test morph‐specific fecundities over the long term, rather than the short term, and should seek to sample natural populations with contrasting female morph frequencies. Experiments such as these on a diverse set of color polymorphism damselflies and other polymorphic species would prove fundamental to help derive a general understanding of morph‐specific fecundities in the maintenance of color polymorphism in animals.

## CONFLICT OF INTEREST

The authors have no conflict of interest to declare.
