# Supplementary material for: Alternative reproductive strategies and the maintenance of female color polymorphism in damselflies
Source: Ecol Evol. 2017 Jun 15;7(15):5592–602. doi: 10.1002/ece3.3083 (PMC5552903; doi:10.1002/ece3.3083)
Supplement: Supplementary file 1 [file ECE3-7-5592-s001.docx]

**Table S1.** Summary of model selection statistics (AIC values, ΔAIC and AIC Weight) of the effects of population (P), female morph (M), and year (Y) on male behavioral response (approximation, attempt to tandem or tandem) to females. For models that gained similar support (ΔAIC < 2), we selected the model with the fewest number of parameters as the most parsimonious model (Burnham and Anderson 2002). The selected model is indicated in bold.

|  | General Lineal Model | AIC | ΔAIC | AIC Weight |
| --- | --- | --- | --- | --- |
| **14** | **M + Y** | **392.82** | **0** | **0.38** |
| 8 | P+M+Y | 394.25 | 1.43 | 0.19 |
| 12 | P + M | 394.28 | 1.46 | 0.18 |
| 6 | P + M + Y + (P x Y) | 396.18 | 3.36 | 0.07 |
| 9 | P + M + (P x M) | 396.43 | 3.61 | 0.06 |
| 5 | P + M + Y + (P x M) | 397.13 | 4.31 | 0.04 |
| 7 | P + M + Y + (M x Y) | 397.47 | 4.65 | 0.04 |
| 2 | P + M + Y + (P x M) + (P x Y) | 399.17 | 6.35 | 0.02 |
| 4 | P + M + Y + (P x Y) + (M x Y) | 399.47 | 6.65 | 0.01 |
| 3 | P + M + Y + (P x M) + (M x Y) | 400.88 | 8.06 | 0.01 |
| 1 | P + M + Y + (P x M) + (P x Y) + (M x Y) | 402.88 | 10.06 | 0 |
| 17 | Y | 409.09 | 16.27 | 0 |
| 13 | P + Y | 409.62 | 16.8 | 0 |
| 16 | M | 409.95 | 17.13 | 0 |
| 10 | P + Y + (P x Y) | 412.72 | 19.92 | 0 |
| 15 | P | 421.04 | 28.22 | 0 |
| 11 | M + Y + (M x Y) | 496.43 | 103.61 | 0 |
