# Supplementary material for: Alternative reproductive strategies and the maintenance of female color polymorphism in damselflies
Source: Ecol Evol. 2017 Jun 15;7(15):5592–602. doi: 10.1002/ece3.3083 (PMC5552903; doi:10.1002/ece3.3083)
Supplement: Supplementary file 2 [file ECE3-7-5592-s002.docx]

**Table S2.** Summary of model selection statistics (AIC values, ΔAIC and AIC Weight) of population (P) and female morph (M), and on non-sexual responses towards males (*time hidden*, *no-response*, *spread and curl abdomen*, *move around the perch*, *fly-away*, *charge* and *face-off*), sexual responses (*attempt to tandem*, *tandem* and *mating*). The selected model is indicated in bold.

|  | **AIC** | | | | **ΔAIC** | | | | **AIC Weight** | | | |
| --- | --- | --- | --- | --- | --- | --- | --- | --- | --- | --- | --- | --- |
| **Behaviour** | **P + M +**  **(P x M)** | **P + M** | **P** | **M** | **P + M +**  **(P x M)** | **P + M** | **P** | **M** | **P + M +**  **(P x M)** | **P + M** | **P** | **M** |
| *Time hidden* | 1035.25 | **1031.77** | 1040.54 | 1038.21 | 3.48 | **0.00** | 8.77 | 6.44 | 0.14 | **0.81** | 0.01 | 0.03 |
| *No response* | 242.18 | 238.03 | 237.33 | **234.88** | 7.30 | 3.15 | 2.45 | **0.00** | 0.02 | 0.14 | 0.19 | **0.65** |
| *Spread* | 535.45 | 532 | 533.29 | **528.63** | 6.82 | 3.37 | 4.66 | **0.00** | 0.03 | 0.14 | 0.07 | **0.76** |
| *Move around the perch* | 395.77 | 391.52 | **390.6** | 393.48 | 5.17 | 0.92 | **0.00** | 2.88 | 0.04 | 0.32 | **0.51** | 0.12 |
| *Fly-away* | 473.35 | 470.09 | **467.93** | 469.55 | 5.42 | 2.16 | **0.00** | 1.62 | 0.04 | 0.18 | **0.54** | 0.24 |
| *Charge* | 94.49 | 90.74 | **90.52** | 94.21 | 3.97 | 0.22 | **0.00** | 3.69 | 0.06 | 0.41 | **0.46** | 0.07 |
| *Face-off* | 426.81 | 425.86 | 429.3 | **422.51** | 4.30 | 3.35 | 6.79 | **0.00** | 0.09 | 0.14 | 0.03 | **0.75** |
| *Attempt to tandem* | 131.76 | 130.17 | **129.19** | 151.18 | 2.57 | 0.98 | **0.00** | 21.99 | 0.15 | 0.32 | **0.53** | 0.00 |
| *Tandem* | 46.17 | 43.8 | **42.53** | 44.83 | 3.64 | 1.27 | **0.00** | 2.30 | 0.08 | 0.26 | **0.50** | 0.16 |
| *Mating* | 35.93 | 32.91 | **30.91** | 33.73 | 5.02 | 2.00 | **0.00** | 2.82 | 0.05 | 0.22 | **0.59** | 0.14 |
